# Genome Sequence of an Unknown Subtype of Hepatitis C Virus Genotype 6: Another Piece for the Taxonomic Puzzle

**DOI:** 10.1128/MRA.01030-19

**Published:** 2019-10-17

**Authors:** Martin Schou Pedersen, Sarah Mollerup, Lone Gilmor Nielsen, Håvard Jenssen, Jens Bukh, Kristian Schønning

**Affiliations:** a Department of Clinical Microbiology, Copenhagen University Hospital, Hvidovre, Denmark; b Department of Science and Environment, Roskilde University, Roskilde, Denmark; c Copenhagen Hepatitis C Program (CO-HEP), Department of Infectious Diseases, Copenhagen University Hospital, Hvidovre, Denmark; d Department of Immunology and Microbiology, Faculty of Health and Medical Sciences, University of Copenhagen, Copenhagen, Denmark; e Department of Clinical Medicine, Faculty of Health and Medical Sciences, University of Copenhagen, Copenhagen, Denmark; Portland State University

## Abstract

The surveillance and correct subtyping of hepatitis C virus strains require available and up-to-date publicly available reference genomes. Here, we present the complete open reading frame sequence of a hepatitis C virus genotype 6 strain of an unknown subtype that was discovered during routine subtyping of patients in the clinic.

## ANNOUNCEMENT

Hepatitis C virus (HCV) is a worldwide pathogen that belongs to the genus *Hepacivirus* within the *Flaviviridae* family. The viral genome is positive-sense, single-stranded RNA, approximately 9,600 nucleotides long, with a single open reading frame (ORF) about 9,000 nucleotides long (1). There are 8 recognized main variants of HCV (genotypes 1 to 8) with up to 35% nucleotide divergence and 90 accepted subtypes deviating up to 25% (2). Genotype 6, the most diverse of the genotypes, is most commonly observed in Asia (3) and continues to be expanded with novel subtype sequences (4). The International Committee on Taxonomy of Viruses (ICTV) requires 3 independent isolates to accept a new subtype (2). Here, we present the HCV ORF sequence of a yet-to-be-defined subtype identified from a patient sample (HVH-HCV334) in January 2019 in Copenhagen, Denmark, during routine analyses.

**FIG 1:**
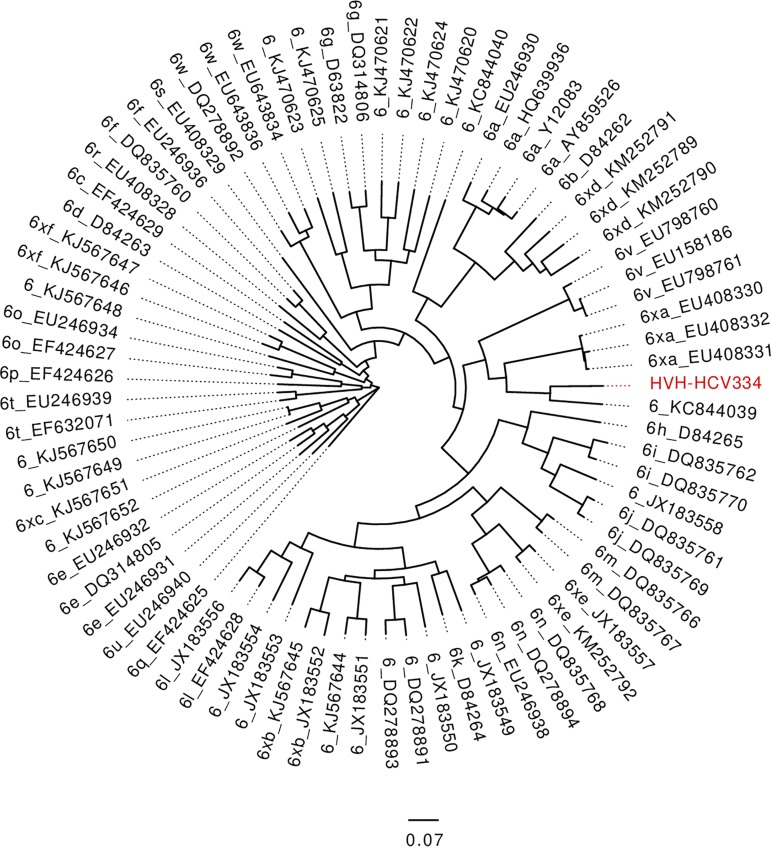
Phylogenetic tree with International Committee on Taxonomy of Viruses (ICTV) genotype 6 reference samples and genotype 6 samples without a designated subtype according to the ICTV classification. Sequences identified as genotype 6 according to the ICTV classification were obtained from NCBI GenBank. Samples were aligned with MUSCLE v.3.8.1551; a maximum likelihood phylogenetic tree was created with FastTree v.2.1.5 and visualized in FigTree v.1.4.3. Branch labels show the designated subtypes and NCBI GenBank accession numbers for the individual samples. HVH-HCV334 is colored red.

The sample had a viral load of 7.51 log IU/ml, as measured by the Aptima HCV Quant Dx assay (5). RNA was extracted with the ZR viral RNA kit (Zymo Research) as described (6) and depleted for human rRNA with the NEBNext rRNA depletion kit (New England BioLabs). RNA sequencing (RNA-seq) libraries were prepared with the NEBNext Ultra II directional RNA library prep kit (New England BioLabs) in half the standard reaction volume suggested by the manufacturer. Sequencing was performed with 2 × 150-bp reads on a MiSeq instrument (Illumina). All software was used with default parameters unless specified. Reads (∼4.5 million) were trimmed and quality filtered with fastp v.0.12.2 (7) to retain a Phred quality of >20 and reads of >50 bp. Filtered reads (∼4 million) were mapped to the human genome hg38 (GenBank accession no. GCA_000001405.27) with Bowtie2 v.2.3.4.1 (8), and unmapped reads (∼2.5 million) were sorted by SAMtools v.1.9 (9), extracted with BEDtools v.2.26.0 (10), and assembled with VICUNA v.1.3 (11). The HCV ORF was identified and annotated with Geneious v.10.2.3 (12) based on reference strain H77 (GenBank accession no. NC_004102). The ORF was 9,069 nucleotides long, without premature stop codons, and annotation identified 3 structural proteins and 7 nonstructural proteins flanked by incomplete 5′ and 3′ untranslated regions. No recombination or subgenomic deletion variants were observed by previously described methods (6). The Geneious statistics function reported a depth of coverage of ∼24,500 and a G+C content of 56%. All official genotype 6 references and sequences without subtype assignment according to the International Committee on Taxonomy of Viruses (ICTV) classification from May 2019 (2) and HVH-HCV334 were aligned with MUSCLE v.3.8.1551 (13); a maximum likelihood phylogenetic tree was created with FastTree v.2.1.5 (14) and visualized in FigTree v.1.4.3 (http://tree.bio.ed.ac.uk/software/figtree/). As seen in Fig. 1, HVH-HCV334 was located close to the untyped sample with GenBank accession no. KC844039 (15) from China and had 81% pairwise nucleotide identity across the ORF. Possible resistance toward ombitasvir, and thus potentially other NS5A inhibitors, was predicted, by HCV GLUE v.0.1.58 (16), due to the resistance-associated amino acids M28 (99%) and S93 (99%) in the NS5A protein (17, 18).

This new genotype 6 genome sequence is important for accurate characterization of HCV for surveillance and prior to antiviral treatment with nonpangenotypic regimens.

### Data availability.

The sequencing reads have been deposited at NCBI under BioProject no. PRJNA557264, and the genome sequence has been deposited in GenBank under accession no. MN240359.
